# Acute Portal Vein Thrombosis Diagnosed with Point-of-care Ultrasonography

**DOI:** 10.5811/cpcem.2016.11.32979

**Published:** 2017-01-24

**Authors:** Daniel Wells, Abigail Brackney

**Affiliations:** * Henry Ford Medical Center - Fairlane, Department of Emergency Medicine, Dearborn, Michigan; † Beaumont Hospital – Royal Oak, Department of Emergency Medicine, Royal Oak, Michigan

## Abstract

Abdominal pain is the most common presenting complaint to the emergency department (ED);1 however, acute portal vein thrombosis is an uncommon cause of abdominal pain. In the following case report, we present a patient who presented to the ED with symptoms of gastroenteritis but was ultimately diagnosed with acute portal vein thrombosis by point-of-care ultrasound (POCUS).

## INTRODUCTION

Abdominal pain is the most common presenting complaint to the emergency department (ED) according to the National Hospital Ambulatory Medical Care Survey, with 11.1 million visits per year in 2011 with the highest rate among females age 25–44.^1^ There are many different etiologies for abdominal pain and the job of the emergency physician is to differentiate serious and life-threatening causes from more benign etiologies. Portal vein thrombosis (PVT) is an uncommon cause of abdominal pain in the ED.^2^ Most PVTs found are chronic and are an incidental finding on computed tomography (CT) or Doppler ultrasonography. However, PVTs can also occur acutely and usually present with more symptoms than chronic PVTs. In the following case report we present a patient who was diagnosed with acute PVT by point-of-care ultrasound (POCUS), and on further imaging was found to have extension into the superior mesenteric vein with subsequent ischemic small bowel.

## CASE REPORT

**Image 1:**
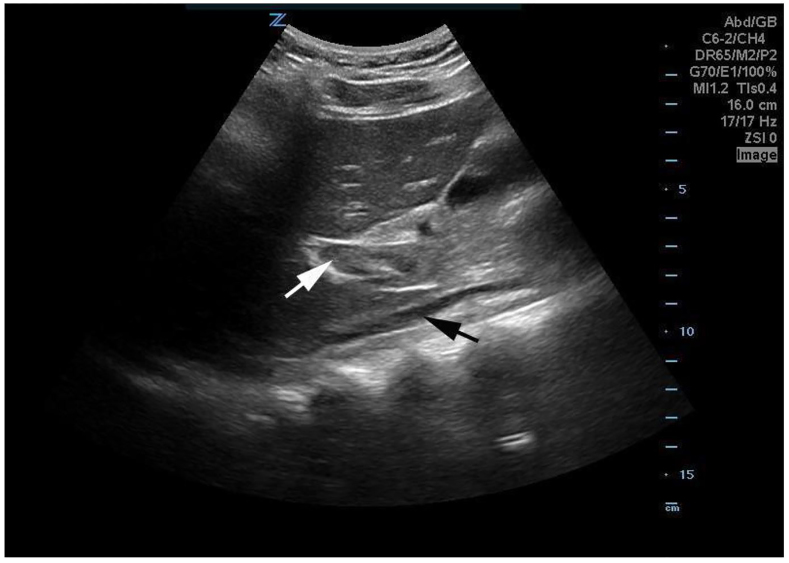
Subcostal oblique parasagittal gray-scale image demonstrates an echogenic focus in the portal vein (white arrow), consistent with clot and a small inferior vena cava (black arrow). *IVC,* inferior vena cava

**Image 2:**
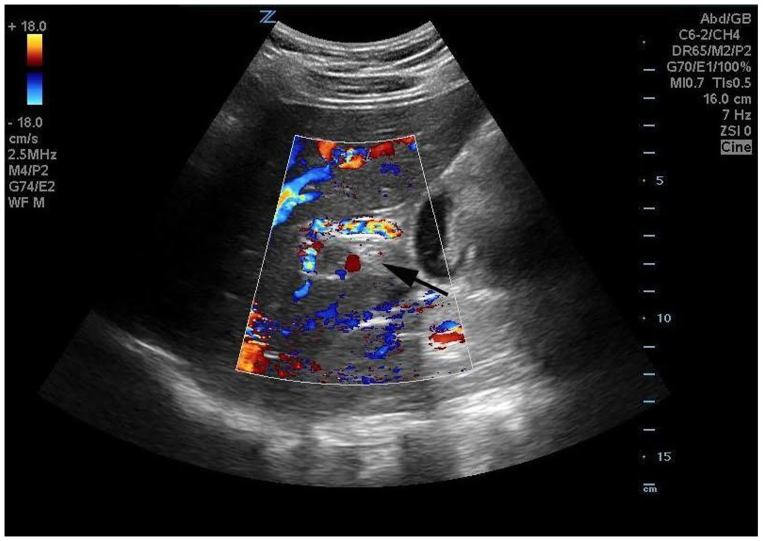
Subcostal oblique parasagittal image with color flow Doppler demonstrates a lack of color flow in the portal vein (black arrow).

**Image 3:**
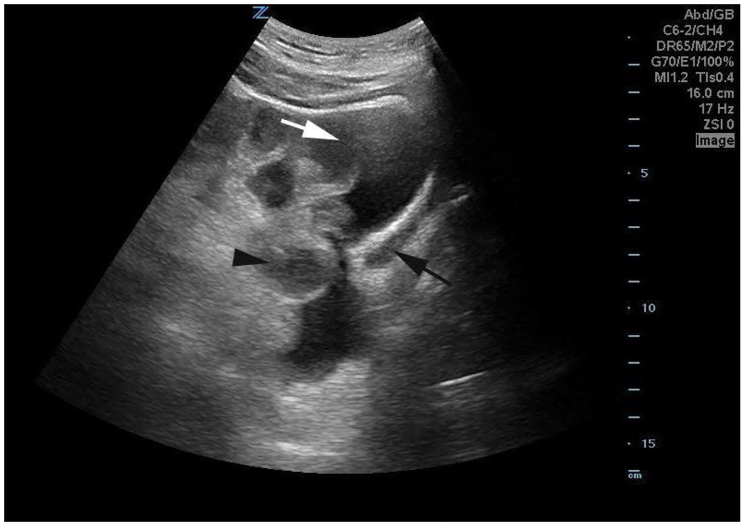
Sagital suprapubic image demonstrates free fluid within the pelvis (white arrow) with a small bladder (black arrow) and thickened bowel (black arrow head), concerning for ischemic bowel.

A 36-year-old female presented to the ED complaining of five days of nausea and vomiting. She developed diarrhea one day prior to her visit and had acutely worsening diffuse abdominal discomfort. She had been seen in clinic four days prior and was diagnosed with suspected viral gastroenteritis. She was given ondansetron and encouraged to drink fluids to rehydrate. The patient denied smoking, and consumed alcohol occasionally. Her only outpatient medications were cetirizine for seasonal allergies and levonorgestrel-ethinyl for contraception. Upon presentation her blood pressure was 134/100mmHg, she was tachycardic (110 beats/minute), tachypneic (29 breaths/minute) and afebrile (97.5rile On exam she was noted to have mild diffuse abdominal tenderness without rebound or guarding, and there was no hepatosplenomegaly. The patient’s abdomen was not distended, there was no fluid wave concerning for ascites or distended abdominal wall vessels. Initial laboratory data showed an aspartate aminotransferase (AST) of 116U/L and alanine aminotransferase (ALT) of 267U/L. Her alkaline phosphatase was unremarkable at 96 U/L. Total bilirubin was 1.8mg/dL. Her lipase was unremarkable. She had marked leukocytosis with WBC of 21.6 bil/L and left shift with neutrophils 15.4 bil/L, and a lactate of 3.1 mmol/L. Given the elevation in liver function tests, a POCUS was performed to investigate for gallbladder pathology using a Zonare C6-2 curved array probe. The POCUS showed free fluid in the abdomen and bowel wall thickening, as well as an echogenic focus in the portal vein without color flow consistent with PVT (Images 1–3). The patient was taken directly for computed tomography (CT) instead of a more comprehensive radiology-performed ultrasound, as she appeared to have complications secondary to the PVT given the abnormal appearance of her bowel on POCUS. The CT showed a small amount of perihepatic ascites, a large filling defect within the portal vein and diffuse small-bowel wall edema and mild dilation of the duodenum suspicious for ischemia.

General surgery was consulted, heparin was initiated, and fluid resuscitation was continued with a resultant decrease in her lactate to 2.2 mmol/L. The patient was admitted to the surgical intensive care unit with plan for surgery the following day. A radiology-performed ultrasound confirmed the PVT, with extension into the proximal splenic vein. Despite anticoagulation and aggressive intravenous fluid resuscitation, the patient’s condition deteriorated overnight and she was noted to have an increasing lactate to 3.7 mmol/L. She was subsequently taken emergently to the operating room for an exploratory laparotomy. Interventional radiology was present and performed a mechanical and pharmacologic thrombectomy. A 35 cm area of ischemic small bowel was then resected and anastomosis was performed two days later. The patient was discharged 12 days after initial presentation on warfarin and off oral contraceptives. Initial hypercoagulability workup showed elevated anticardiolipin IgM titers suspicious for anticardiolipin antibody syndrome; however, the confirmatory tests were negative.

## DISCUSSION

PVT is an uncommon cause of abdominal pain, with an overall incidence of 1%.^2^ It is most often a chronic condition that is found incidentally in the ED with Doppler ultrasonography or CT. However, PVT can also occur acutely. Acute presentations are normally symptomatic with patients complaining of abdominal pain, diarrhea and ileus. Common complications of PVT include ischemic hepatitis, variceal bleeding and, infrequently, small bowel ischemia.^3^

Due to the rarity of PVT there are few large studies exploring its complications and risk factors. The incidence, however, is known to increase with a number of conditions, most significantly cirrhosis and hepatobiliary malignancy. Other factors that increase the risk of PVT include abdominal infections, inflammatory disease, myeloproliferative disorders, coagulopathies and use of oral contraceptives.^2^,^4^

The presentation, diagnosis and associated complications of PVT differ based on whether the clot is acute or chronic, isolated to the portal vein, or if there is extension into the mesenteric or splenic veins. Isolated PVT,is often asymptomatic and has been found to rarely cause acute ischemia. In one study only 39% of patients with isolated PVT reported symptoms, most commonly abdominal pain. Patients with extension of the clot into the mesenteric vein reported symptoms in 92% of cases and 45% were found to have intestinal infarction.^4^ Patients with acute PVT are also more likely to complain of nonbloody diarrhea. They often have small-volume ascites, systemic inflammatory response syndrome and peritonitis. Although patients with chronic PVT can develop ischemia, they are more likely to present with hematemesis or melena due to the development of portal hypertension and variceal hemorrhage.^3^

The percentage of patients with PVT with extension into the mesenteric vein has been estimated to be as high as 55%.^5^ Presence of thrombosis in the mesenteric vein was also associated with higher mortality rates, estimated from 20 to 50%.^6^ The duration of the thrombosis in the mesenteric vein also affects mortality rates. Patients with acute clots have a three-year survival rate of 36% versus 83% for those with chronic thromboses.^7^ Isolated mesenteric venous thrombosis, without concurrent portal vein clot, have also been reported. Patients presenting with isolated mesenteric thrombosis are a particular challenge to clinicians as both ultrasound and CT, with combined sensitivity of only 50%, often fail to visualize the clot as compared to a 97% combined sensitivity when the thrombosis extends into the portal or splenic vein. If there is high suspicion, angiography remains the gold standard for diagnosis.^8^

Ultrasound has been used to diagnose PVT for decades, but given that up to 30% of patients with PVT will not have an echogenic thrombosis on gray-scale US it was not until the advent of color Doppler imaging that it became a reliable test.^9^ With color Doppler, clinicians are able to visualize flow noninvasively. When compared to angiography or surgery, color Doppler US has a sensitivity of 89% and specificity of 92%. 9 Characteristics of PVT on color Doppler include increased arterial flow in patient with low portal flow, postprandial reversal of portal flow from hepatopetal to hepatofugal, and development of collateral circulation.^10^ Acute and chronic thrombosis appear differently with color Doppler. In both cases an echogenic thrombus is often but not universally seen. Chronic PVT more often will show cavernous transformation of the portal vein with collateral formation.^9^,^10^

Both acute and chronic PVTs are typically treated with oral anticoagulants. However, if there is extension of the clot into the mesenteric vein with evidence of ischemia, more invasive measures, including surgery and thrombectomy, may be indicated. This case shows the importance of keeping PVT on the differential for patients presenting with abdominal pain. It also shows that POCUS with color Doppler may speed the diagnosis of this potential life-threatening condition.
